# Peroxidase-Mimicking Nanozymes of Nitrogen Heteroatom-Containing Graphene Oxide for Biomedical Applications

**DOI:** 10.3390/bios15070435

**Published:** 2025-07-07

**Authors:** Phan Gia Le, Daesoo Kim, Jae-Pil Chung, Sungbo Cho

**Affiliations:** 1 Department of Electronic Engineering, Gachon University, Seongnam-si 13120, Republic of Korea; 2 Department of Semiconductor Engineering, Gachon University, Seongnam-si 13120, Republic of Korea; 3 Department of Health Sciences and Technology, Gachon University, Incheon 21999, Republic of Korea

**Keywords:** nanozyme, graphene oxide, peroxidase, HRP, biosensors

## Abstract

Nanozymes constitute a rapidly advancing frontier in scientific research, attracting widespread international interest, particularly for their role in facilitating cascade reactions. Despite their initial discovery a few years ago, significant hurdles persist in optimizing their catalytic performance and substrate specificity—challenges that are especially critical in the context of biomedical diagnostics. Within this domain, nitrogen-containing graphene oxide-based nanozymes exhibiting peroxidase-mimicking activity have emerged as particularly promising candidates, owing to the exceptional electrical conductivity, mechanical flexibility, and structural resilience of reduced graphene oxide-based materials. Intensive efforts have been devoted to engineering graphene oxide structures to enhance their peroxidase-like functionality. Nonetheless, the practical implementation of such nanozymes remains under active investigation and demands further refinement. This review synthesizes the current developments in nitrogen heteroatom-containing graphene oxide nanozymes and their derivative nanozymes, emphasizing recent breakthroughs and biomedical applications. It concludes by exploring prospective directions and the broader potential of these materials in the biomedical landscape.

## 1. Introduction

Nanozymes—nanomaterials exhibiting enzyme–mimetic catalytic properties—have emerged as a rapidly expanding area of research in recent years [1,2]. Their potential is particularly significant in biomedical contexts, where they can functionally substitute natural enzymes in cascade reactions. The foundational concept of nanozymes dates back to 2004, when Manea et al. immobilized triazacyclononane/Zn^2+^ complexes on gold nanoparticles to facilitate phosphodiester bond cleavage, demonstrating superior catalytic performance compared to their free counterparts [3]. In 2006, Lévy et al. advanced this concept by mimicking protein structures through the immobilization of peptides on gold nanoparticles [4]. The term “nanozyme” gained broader recognition in 2007, appearing in three influential publications. One such study, following the work of Manea and the Scrimin group, focused on protein mimicry [5]; Pluth et al. introduced a Ga^3+^-containing supramolecular assembly classified as a nanozyme [6]; and Batrakova et al. described a nanozyme formed by encapsulating catalase within a polyethyleneimine–poly (ethylene glycol) diblock polymer matrix [7]. That same year, Gao et al. reported the peroxidase-like activity of Fe_3_O_4_ nanoparticles, marking a pivotal moment in nanozyme research by clearly articulating the concept through a comprehensive analysis of their physicochemical characteristics [8]. This breakthrough has since catalyzed a surge of studies focused on peroxidase-mimicking nanozymes, laying the groundwork for extensive exploration in this domain.

**Figure 1:**
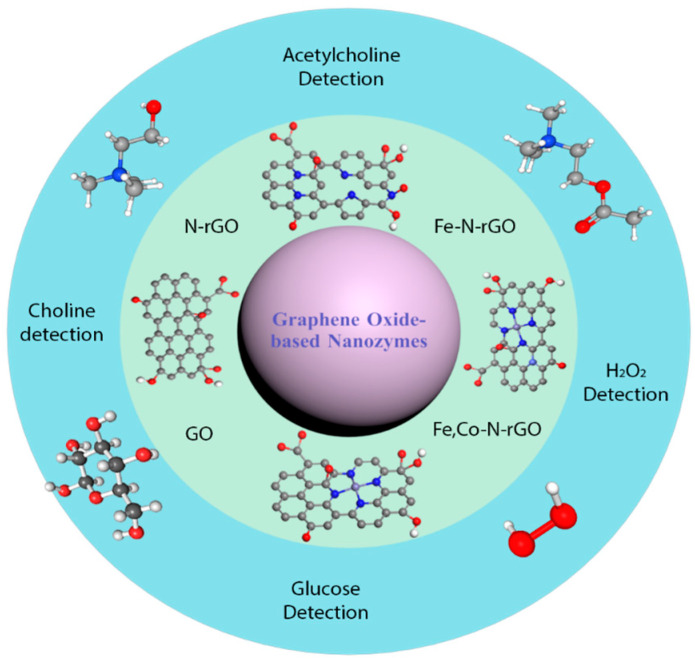
Overview of graphene oxide-based nanozymes and their biomedical application.

To date, six primary classes of catalytic enzymes have been identified [9], of which four—oxidoreductases [10,11], hydrolases [12,13], lyases [14], and isomerases [15]—have been researched, encompassing over thirty distinct enzyme–mimetic types [16]. Among these, oxidoreductase-like nanozymes have received the most extensive investigation, largely due to their intrinsic catalytic relevance and the foundational insights provided by earlier studies [8]. A prominent example is the peroxidase-mimicking nanozyme, which is typically evaluated using colorimetric assays that exploit its ability to catalyze the reduction of hydrogen peroxide (H_2_O_2_), leading to a visible color change similar to that produced by horseradish peroxidase (HRP) [17]. Although natural peroxidase enzymes exhibit high substrate specificity and catalytic efficiency, their application is limited by high production costs, purification challenges, and vulnerability to denaturation under harsh environmental conditions [18,19]. To overcome these limitations, considerable research efforts have been directed toward the development of peroxidase-like nanozymes as robust and cost-effective alternatives for use in cascade reactions relevant to biomedical and environmental applications [20]. Various classes of materials have been explored for their peroxidase-like activity, including metals [21,22], metal oxides [23,24], carbon-based materials [25,26], metal–organic frameworks (MOFs) [27,28], MXenes [29,30], composites [31,32], and organic–inorganic hybrids [33]. Among these, carbon-based nanozymes—particularly those derived from graphene oxide—have attracted substantial attention due to their unique physicochemical properties and the structural tunability that enables broad applicability across sectors such as environmental monitoring, food safety, healthcare, and clinical diagnostics [34,35]. Graphene oxide-based nanozymes have been engineered either by modifying the pristine graphene oxide structure [36,37] or by forming composites with other materials [38,39]. In general, these graphene oxide-based nanozymes exhibit enhanced stability under extreme pH and temperature conditions compared to natural enzymes [20,26]. However, certain composite materials have shown reduced stability under varying pH conditions. A schematic overview of graphene oxide-based peroxidase-mimicking nanozymes is presented in Figure 1.

With ongoing advancements in technology, the design and prediction of nanozyme models have increasingly leveraged high-performance computing systems [40] and artificial intelligence (AI) algorithms [41]. These computational approaches significantly reduce the time and labor associated with experimental procedures. However, their predictive accuracy remains constrained by inherent approximations and the absence of real-world complexities. Nevertheless, computational methods, such as density functional theory (DFT) [42], molecular docking simulations [43], and molecular dynamics (MD) [44], offer valuable insights by enabling extrapolation from theoretical principles, often providing perspectives that complement or precede experimental findings.

In this review, we present a comprehensive overview of graphene oxide-based peroxidase-mimicking nanozymes. Particular emphasis is placed on recent advances in nitrogen-doped graphene oxide nanozymes, synthesized through structural modification of graphene oxide, with illustrative examples focused on biomedical applications. Finally, we discuss future directions and the promising potential of these nanozymes for expanded use in biomedical fields.

## 2. Overview of the Graphene Oxide, Its Derivatives, and Peroxidase-like Activity

### 2.1. Overview of Graphene Oxide

Graphene oxide (GO) can be synthesized through both bottom-up and top-down methodologies, including well-established techniques, such as the Hummers [45], Staudenmaier [46], Hofmann [47,48], and Brodie [49,50] methods. However, the physicochemical characteristics of graphene oxide vary significantly depending on the synthesis method employed.

**Figure 2:**
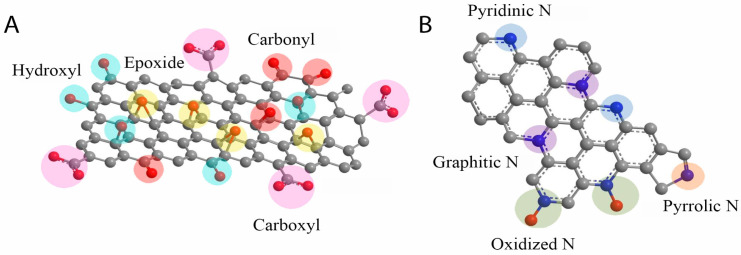
(**A**) Graphene oxide and (**B**) N-doped graphene oxide structures [61].

Structurally, graphene oxide is a two-dimensional (2D) material composed of a single layer of aromatic carbon atoms arranged in a hexagonal lattice with sp^2^ hybridization. Covalent bonding within the lattice is accompanied by the presence of oxygen-containing functional groups anchored to the surface. GO exhibits a wide band gap ranging from 1.1 to 4.7 eV, making it suitable for various electronic and optical applications [51]. The degree of structural disorder and the nature of defects within the sp^2^ carbon domain can be analyzed using Raman spectroscopy, primarily through the D and G bands located at approximately 1350 cm^−1^ and 1590 cm^−1^, respectively [52,53]. The intensity ratio of the D to G bands (I_D_/I_G_) serves as a reliable indicator for assessing the extent of disorder and the reduction level of GO, as well as distinguishing between pure and impure forms [54,55]. Additionally, the presence of a broad, weak 2D peak near 2650 cm^−1^ provides insight into the number of graphene oxide layers, although it is not clear like the graphene case. The position, shape, and intensity of the 2D Raman peak are influenced by both the number of layers and the degree of oxidation. In comparison to pristine graphene, the 2D peak of graphene oxide is significantly broader, which can be attributed to the presence of structural defects and oxygen-containing functional groups [56,57]. Beyond its aromatic carbon backbone, GO possesses a rich variety of surface functional groups, including hydroxyl (-OH), carboxyl (-COOH), carbonyl (-C=O), epoxy (C-O-C), and ester (-COOC-) moieties, all of which significantly influence its physicochemical behavior [58,59,60], as depicted in Figure 2A. The modification or removal of these functional groups through physical or chemical treatments enables the tailoring of graphene oxide properties for specific applications.

Graphene oxide (GO) serves as an ideal substrate for the facile doping of heteroatoms such as nitrogen (N), boron (B), phosphorus (P), sulfur (S), and various transition metals (M or M’) [26,34,62,63]. The incorporation of these heteroatoms into the GO lattice induces a delocalization of electronic states, thereby significantly altering its physicochemical properties.

To enhance the electrical conductivity of graphene oxide, various physicochemical modification techniques have been employed. These treatments effectively reduce the bandgap, elevate the Fermi level, and promote the transition of electrons from the valence to the conduction band. Reduced graphene oxide (rGO), characterized by superior physicochemical attributes, has been extensively utilized across diverse research domains, including energy storage, sensing technologies, healthcare, and wearable electronics. In biosensor applications, graphene can participate in both direct and indirect chemical interactions, functioning either as an active component or as a supportive matrix for biochemical reactions.

### 2.2. Overview of Peroxidase-like Activity

Horseradish peroxidase (HRP) is a naturally occurring enzyme capable of catalyzing the reduction of hydrogen peroxide (H_2_O_2_), thereby facilitating the oxidation of chromogenic substrates from a colorless state to a distinct coloration. The catalytic mechanism involves the conversion of H_2_O_2_ into hydroxyl radicals (•OH), which subsequently oxidize various chromogenic compounds, such as 3,3′,5,5′-tetramethylbenzidine (TMB), 2,2′-azino-bis (3-ethylbenzthiazoline-6-sulfonic acid) (ABTS), o-phenylenediamine dihydrochloride (OPD), and oxygen red, resulting in observable color changes [17,34]. Due to their transient nature, hydroxyl radicals can be indirectly detected using terephthalic acid, which captures •OH and forms a fluorescent product measurable at a wavelength of 425 nm via fluorescence spectroscopy [20]. Nanozymes with peroxidase-like activity exhibit catalytic behaviors comparable to those of natural enzymes, while offering distinct advantages, including facile synthesis, enhanced stability under extreme conditions (e.g., temperature and pH), and prolonged shelf life. In many cases, the catalytic efficiency of such nanozymes surpasses that of their natural counterparts by several orders of magnitude [26,34].

## 3. Recently Advanced Development and Progression of the N Heteroatom-Containing GO-Based Nanozymes

Graphene oxide serves as a versatile foundational material for engineering high-performance catalytic systems. Structural modifications to graphene oxide, coupled with the incorporation of heteroatoms into its lattice, induce a redistribution of electron density, thereby modulating its physicochemical properties. Common dopants used in this context include nitrogen (N), sulfur (S), phosphorus (P), and boron (B). The introduction of these heteroatoms not only alters the local electronic environment, but also modifies electron transport pathways by creating active sites on the graphene surface through bridging interactions with adjacent carbon atoms. Among these, nitrogen-doped graphene oxide nanozymes have withdrawn significant attention, both in the context of nitrogen-only doping and in combination with transition metal elements. Additionally, nanozymes doped with S, P, or B are emerging as promising candidates for various biosensor applications due to their tunable electronic structures and catalytic functionalities.

### 3.1. Graphene Oxide Nanozymes and Their Biomedical Application

Song et al. reported the development of a graphene-derived nanozyme, wherein graphene oxide functionalized with carboxyl groups (GO-COOH) was synthesized from graphite and subsequently dispersed in a neutral pH (7.0) solution as the final product [64]. This nanozyme exhibited intrinsic peroxidase-mimicking activity, effectively catalyzing the oxidation of the chromogenic substrate TMB in the presence of hydrogen peroxide (H_2_O_2_). The catalytic process followed a ping-pong (double displacement) reaction mechanism. Utilized in a colorimetric sensing platform, the nanozyme enabled the quantitative detection of H_2_O_2_ and glucose within linear concentration ranges of 5 × 10^−8^–1 × 10^−6^ M and 1 × 10^−6^–2 × 10^−5^ M, respectively, achieving detection limits of 5 × 10^−8^ M for H_2_O_2_ and 1 × 10^−6^ M for glucose. The sensor demonstrates applicability for both healthy individuals and diabetic patients. The peroxidase-mimicking activity of graphene oxide is primarily ascribed to the presence of carboxyl functional groups, which act as catalytic sites for the reduction of H_2_O_2_ [65]. These functional groups, inherently integrated within the graphene oxide framework, endow the material with enzyme-like properties. Nonetheless, the intrinsic peroxidase-like activity exhibited by unmodified oxide graphene is relatively weak and significant. Structural modifications or the chemical functionalization of graphene oxide have been shown to significantly enhance its catalytic performance.

### 3.2. Nitrogen-Doped Graphene Oxide Nanozymes and Their Biomedical Application

#### 3.2.1. Synthesis of N-Doped Graphene Oxide

The incorporation of nitrogen atoms into the GO lattice disrupts the original structure of pristine GO, resulting in the formation of new configurations. Among various heteroatomic dopants, N is particularly favorable due to its atomic radius (~0.56 Å), which closely approximates that of carbon (~0.67 Å) [66]. The N-rGO can be synthesized through multiple approaches, including solid-state reactions, hydrothermal synthesis, pyrolysis, and chemical reduction, utilizing a range of nitrogen-containing precursors such as melamine, ammonia, urea, and organic amines [34,35,36,67]. In the solid-state method, N-rGO is typically obtained by blending GO with melamine, followed by thermal treatment at elevated temperatures (≥600 °C) for durations ranging from 30 min to 1 h [20,61], or by sintering GO in an ammonia-rich atmosphere [34]. In pyrolytic synthesis, GO combined with polyaniline is subjected to pyrolysis at around 1000 °C. For chemical reduction, hydrazine is commonly used to reduce GO while incorporating nitrogen species. The synthesis route significantly influences the resulting material properties due to differences in structural outcomes. The literature reports have confirmed that nitrogen doping in GO can yield a variety of nitrogen functionalities, including pyrrolic-, pyridinic-, graphitic-, and oxidized-N species [26,34]. These distinct configurations can be identified via X-ray photoelectron spectroscopy (XPS), with characteristic binding energies observed at approximately 401.1 eV (graphitic-N), 399.6 eV (pyridinic-N), 398.1 eV (pyrrolic-N), and 403.1 eV (oxidized-N) [20]. The integration of nitrogen into the rGO lattice also serves as a foundational structure for further hybridization with transition metals, enhancing its catalytic capabilities [20,34]. Nevertheless, achieving precise control over both the N content and the specific types of N configurations remains a significant synthetic challenge. A representative illustration of the N-rGO nanozyme structure is provided in Figure 2B.

#### 3.2.2. Peroxidase-like Activity of N-Doped Graphene Oxide and Their Biomedical Application

The peroxidase-mimicking activity of the N-rGO has been extensively characterized in previous studies. These works consistently report that N-rGO exhibits catalytic activity several tens to hundreds of times greater than that of pristine rGO. This enhanced performance is attributed to the unique physicochemical properties conferred by N doping, wherein nitrogen atoms are incorporated into various structural configurations—such as pyridinic, pyrrolic, and graphitic nitrogen—that serve as active catalytic sites on the graphene surface. Owing to its peroxidase-like activity, analogous to that of the HRP enzyme, N-rGO has been widely adopted in biosensor applications, particularly in the development of optical and electrochemical sensors. For instance, Hu et al. synthesized N-rGO via a two-step process, using urea and ammonia as N sources, applied to both GO and mesoporous carbon [67]. The resulting nanozyme demonstrated high peroxidase-like activity for H_2_O_2_ catalysis, with relative catalytic activities 100-fold and 60-fold higher than that of rGO in GO and mesoporous carbon contexts, respectively. Reaction energy profiles, calculated using the DFT, were used to elucidate the underlying catalytic mechanisms. In another study, Liang et al. synthesized N-rGO using a hydrothermal method at 180 °C for 3 h, employing GO and ammonium solution as precursors [68]. The resulting nanozyme enabled H_2_O_2_ detection via a colorimetric sensor within a linear range of 100 µM to 1 mM. Furthermore, the generation of hydroxyl radicals (•OH) from H_2_O_2_ was leveraged for tumor-specific therapeutic applications, achieving a tumor cell lethality rate of up to 87%, at a concentration of 0.4 mg·mL^−1^. Siddiqui et al. also employed a hydrothermal approach, synthesizing N-rGO from graphene oxide and urea at 180 °C for 7 h [36]. The sensor based on this nanozyme utilized fluorescence spectroscopy to detect H_2_O_2_ across a linear range of 1 nM to 1 µM, with an impressive detection limit of 94 pM. Additionally, the same nanozyme demonstrated efficacy in degrading Rhodamine B (RhB). Collectively, these studies underscore the versatility of synthesis methods for N-rGO and reaffirm its robust peroxidase-like activity, which is critical for its application across a wide spectrum of fields, including environmental monitoring, biomedical diagnostics, and targeted therapeutics.

#### 3.2.3. N, B-Co-Doped Graphene Oxide Nanozymes and Their Biomedical Applications

**Figure 3:**
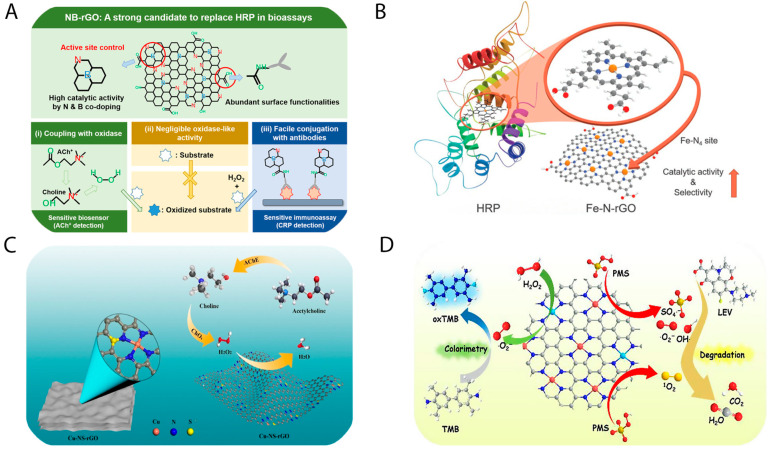
(**A**) N,B-doped graphene oxide nanozyme and their application in detection of choline, acetylcholine, and C-reactive protein [35]; (**B**) Fe-N_4_-C-structured graphene oxide nanozyme mimicking HRP natural enzyme [34]; (**C**) Cu-N_4_- and Cu-N_4_-S-structured graphene oxide nanozyme and its application of choline, acetylcholine detection [26]; (**D**) Fe,Cu-N-structured graphene oxide nanozyme and their application in isoniazid quantification and levofloxacin degradation [71].

As discussed in the previous section, N doping significantly alters the structure of pristine GO, enhancing the catalytic efficiency of nanozymes. Building upon this concept, the incorporation of other non-metallic heteroatoms, such as sulfur (S) and boron (B), has also been shown to further augment the peroxidase-like catalytic performance of GO-based nanozymes. Min Su et al. reported the synthesis of a nitrogen and boron co-doped GO nanozyme via a solid-state reaction method utilizing GO, melamine, and a boron source [35]. The simultaneous incorporation of N and B into the graphene oxide lattice dramatically enhanced its catalytic performance. Specifically, the N,B-co-doped GO demonstrated a 1000-fold increase in catalytic activity for H_2_O_2_ and a 300-fold enhancement for TMB at pH 4, relative to undoped rGO. A colorimetric sensor fabricated from this nanozyme exhibited high sensitivity toward H_2_O_2_, with a linear detection range of 0.5–30 µM and a detection limit of approximately 100 nM. Moreover, the sensor demonstrated excellent performance in detecting biologically relevant analytes such as choline and acetylcholine over the range of 0.05–0.5 µM, with detection limits of ~10 nM and ~30 nM, respectively. Notably, it also enabled the detection of C-reactive protein across a broad concentration range of 1–5000 ng·mL^−1^, with a low detection threshold of ~5 ng·mL^−1^ and a rapid response time of approximately 3 min. To elucidate the underlying catalytic mechanism, the DFT calculations were employed to model the degradation pathway of H_2_O_2_. A schematic representation of the N,B co-doped GO nanozyme is provided in Figure 3A.

In other work, Luo et al. synthesized N,B co-doped GO nanoribbons (N,B-GNRs), utilizing urea and boric acid as nitrogen and boron sources, respectively [69]. The dual doping of N and B introduced a high density of structural defects and catalytically active sites, significantly enhancing the peroxidase-like activity of the N,B-GNRs nanozymes compared to their singly doped counterparts. The resulting sensor exhibited remarkable sensitivity in detecting interleukin-6, achieving a wide linear detection range from 0.001 to 1000 ng/mL and a detection limit as low as 0.3 pg/mL. In a separate study, Li et al. [70] systematically examined the peroxidase-mimetic behavior of N-, B-, and N,B-doped graphene oxides by constructing theoretical reaction models, identifying transition states, and calculating Gibbs free energy changes. Their findings revealed that B-doped graphene oxide outperformed N-doped variants in peroxidase-like activity, while co-doping with both N and B produced a synergistic effect. This enhancement was attributed to increased active site density and electron redistribution, facilitating more efficient electron transfer. These results underscore the effectiveness of non-metallic co-doping strategies in optimizing the catalytic performance of graphene oxide-based nanozymes.

#### 3.2.4. Peroxidase-Mimicking Activity of Metal (M, M’) and N Co-Doped GO Nanozymes and Their Biomedical Applications

The incorporation of N or (N in combination with other non-metallic elements) into the GO lattice induces substantial alterations to its pristine hexagonal structure. Building on this modification, the integration of transition metals further enhances the catalytic architecture by forming M-N_X_-C, M,M’-N_X_-C, or M-S,N_X_-C frameworks. These hybrid configurations not only redistribute electronic density but also modify the electron transfer pathway mechanisms. Transition metals commonly employed for such structures include iron (Fe), copper (Cu), zinc (Zn), and cobalt (Co), leading to the formation of catalytic centers such as Fe-N_4_-C [34], Zn-N_4_-C [20], Co-N_4_-C, etc.

Both computational modeling and experimental validation have demonstrated that the Fe-N_4_ configuration exhibits a lower electron transfer efficiency than Co-N_4_ and Zn-N_4_, which in turn reduces the energy barrier for hydroxyl radical generation, thereby enhancing peroxidase-like activity [72]. In a work by Min Su et al., Fe and N were co-doped into the GO lattice to mimic the Fe-N_4_ coordination environment of natural heme-containing peroxidases (e.g., HRP), as illustrated in Figure 3B [34]. The resulting Fe-N_4_-C nanozyme displayed catalytic activity approximately 700 times greater than that of the rGO. This sensor was capable of detecting choline and acetylcholine within linear ranges of 50–1000 nM, with respective detection limits of 10 nM and 20 nM. Additionally, it could monitor H_2_O_2_ released from cancer cells following stimulation with N-formylmethionyl-leucyl-phenylalanine (fMLP). Further development by Le et al. involved the synthesis of Zn-N_X_-structured graphene oxide, which took into account the defect amount and electron density consideration [20]. This nanozyme demonstrated high sensitivity for H_2_O_2_ detection in sodium acetate buffer, with a linear range of 0.1–10 µM and a detection limit of 1.47 nM. It also functioned effectively as a colorimetric sensor for glucose in human serum (0.25–1.5 mM, detection limit: 0.12 mM). Notably, a paper-based glucose sensor incorporating this nanozyme allowed for smartphone-assisted glucose quantification over a 1–30 mM range, with a detection limit of 0.78 mM—suitable for monitoring both hypoglycemic and hyperglycemic conditions.

At a more advanced level, Le et al. also reported the development of Cu-N_4_S-C and Cu-N_4_-C-structured GO nanozymes [26]. These materials exhibited exceptional peroxidase-like activity, approximately 2500 times greater than rGO, as depicted in Figure 3C. The corresponding electrochemical sensor achieved ultrasensitive detection of H_2_O_2_ within a linear range of 10–1000 fM, and a remarkably low detection limit of ~50 fM. Furthermore, it enabled quantification of choline and acetylcholine in human serum within ranges of 20–200 nM and 20–100 nM, with detection limits of 2.5 nM and 5 nM, respectively, highlighting its diagnostic potential. In another study, Xie et al. synthesized Fe/Cu, N co-doped graphene oxide nanozymes (Figure 3D) [71], which enabled detection of isoniazid across a range of 0.9–10 µM, with a detection limit of 0.3 µM. Additionally, these nanozymes were capable of degrading levofloxacin by up to 90.4% within 30 min. Collectively, these findings demonstrate that M,N- or M,N,S-co-doped graphene oxide nanozymes exhibit significantly superior peroxidase-mimetic activity compared to the rGO. Their high sensitivity in both colorimetric and electrochemical platforms underscores their potential for advanced biomedical application. The N-containing GO-based biosensors have been listed as Table 1.

## 4. Conclusions and Future Perspective

The peroxidase-mimetic activity of N-rGO is substantially enhanced in the presence of additional N, non-metallic, and metallic co-dopants. Such engineered nanozymes hold considerable significance due to their broad applicability across diverse research domains. Nonetheless, achieving precise regulation of N content and the specific N configurations within the graphene oxide matrix remains a formidable challenge. Compared to S-, P-, and B-doped graphene oxide nanozymes, N-doped variants are more extensively investigated. Moreover, graphene oxide nanozymes co-doped with metal and nitrogen (M,N) or dual metals and nitrogen (M,M’,N) exhibit a superior catalytic performance, surpassing that of other doped GO nanozyme analogues by markedly enhancing peroxidase-like activity.

In parallel with technological advancements, numerous enzyme models have been developed and computationally optimized through the use of high-performance computing, machine learning, and deep learning techniques. The design and synthesis of novel nanozymes exhibiting enhanced peroxidase-like properties represent a significant breakthrough in the nanozyme research arena, with promising implications for biomedical applications.

These classes of nanozymes are amenable to large-scale production, particularly via solid-state reactions, albeit with a trade-off in terms of structural uniformity. Moreover, parameters such as dosage and particle size are critical for success in vivo applications. For instance, in the case of graphene oxide-based nanozymes, administering a dose at a low concentration can mitigate potential toxicity, while low particle sizes facilitate effective biological penetration. However, their catalytic performance may be significantly impaired in complex biological matrices such as whole blood, underscoring the need for high substrate specificity and minimized interference effects.

## Figures and Tables

**Table 1:** N-containing GO-based peroxidase-mimicking nanozymes and their biomedical applications.

Nanozymes	Sensor Types	Application	Targets	Environments	Linear Ranges (M)	Detection Limits (M)	Ref.
GO-COOH	Colorimetric sensor	Diabetic mellitus	H_2_O_2_	Phosphate buffer	5 × 10^−8^–10^−6^	5 × 10^−8^	[64]
			Glucose		10^−6^–2 × 10^−5^	10^−6^	
N-rGO	Colorimetric sensor	Tumor treatment	H_2_O_2_	KH_2_PO_4_/Na_2_HPO_4_	10^−4^–10^−3^	NA **	[68]
N-rGO	Colorimetric sensor	Decomposition of Rhodamine B	H_2_O_2_	PBS *	10^−9^–10^−6^	94 × 10^−12^	[36]
N, B-rGO	Fluorescent sensor	Neurotransmitter	H_2_O_2_	Tris-acetate buffer	5 × 10^−7^–3 × 10^−5^	10^−7^	[35]
			Choline		5 × 10^−8^–5 × 10^−6^	10^−8^	
			Acetylcholine		5 × 10^−8^–5 × 10^−6^	3 × 10^−8^	
		Inflammatory marker	C-reactive protein	Bovine serum albumin	1–5000 ng.ml^−1^	5 ng.ml^−1^	
N,B-rGO	Colorimetric sensor	Cytokine detection	Interleukin-6	PBS	0.001–1000 ng.ml^−1^	0.3 pgmL^−1^	[69]
Fe-N_4_-C	Colorimetric sensor	Neurotransmitter	Choline	Tris-acetate buffer	5 × 10^−8^–10^−6^	10^−8^	[34]
			Choline	Tris-acetate buffer	5 × 10^−8^–10^−6^	2 × 10^−8^	
			Acetyl Choline	Tris-acetate buffer	5 × 10^−9^–10^−7^	2 × 10^−8^	
Cu-N_4_ and Cu-N_4_-S	Electrochemical sensor	Neurotransmitter	H_2_O_2_	PBS	10^−14^–10^−12^	5.0 × 10^−14^	[26]
			Choline	Human serum	10^−9^–10^−7^	2.5 × 10^−9^	
			Acetyl Choline	Human serum	2 × 10^−8^–10^−7^	5.0 × 10^−9^	
Zn-N_4_-C	Colorimetric sensor	Diabetic Mellitus	H_2_O_2_	PBS	10^−7^–10^−6^	1.47 × 10^−9^	[20]
	Colorimetric sensor		Glucose	Human serum	2.5 × 10^−4^–1.5 × 10^−3^	0.12 × 10^−3^	
	Glucose paper chip		Glucose	Human serum	10^−3^–3 × 10^−2^	0.78 × 10^−3^	
FeCu-NC	Colorimetric sensor	Antibiotic detection	Isoniazid	Sodium Acetate buffer	9 × 10^−7^–10^−5^	3 × 10^−7^	[72]

(*): Phosphate buffer saline. (**): not available.

## Data Availability

Not applicable.

## References

[1] Huang Y., Ren J., Qu X. (2019). Nanozymes: Classification, Catalytic Mechanisms, Activity Regulation, and Applications. Chem. Rev..

[2] Liang M., Yan X. (2019). Nanozymes: From New Concepts, Mechanisms, and Standards to Applications. Acc. Chem. Res..

[3] Manea F., Houillon F.B., Pasquato L., Scrimin P. (2004). Nanozymes: Gold-Nanoparticle-Based Transphosphorylation Catalysts. Angew. Chem. Int. Ed..

[4] Lévy R. (2006). Peptide-Capped Gold Nanoparticles: Towards Artificial Proteins. ChemBioChem.

[5] Pengo P., Baltzer L., Pasquato L., Scrimin P. (2007). Substrate Modulation of the Activity of an Artificial Nanoesterase Made of Peptide-Functionalized Gold Nanoparticles. Angew. Chem. Int. Ed..

[6] Pluth M.D., Bergman R.G., Raymond K.N. (2007). Acid Catalysis in Basic Solution: A Supramolecular Host Promotes Orthoformate Hydrolysis. Science.

[7] Batrakova E.V., Li S., Reynolds A.D., Mosley R.L., Bronich T.K., Kabanov A.V., Gendelman H.E. (2007). A Macrophage–Nanozyme Delivery System for Parkinson’s Disease. Bioconjugate Chem..

[8] Gao L., Zhuang J., Nie L., Zhang J., Zhang Y., Gu N., Wang T., Feng J., Yang D., Perrett S. (2007). Intrinsic peroxidase-like activity of ferromagnetic nanoparticles. Nat. Nanotechnol..

[9] Le P.G., Kim M.I. (2021). Research Progress and Prospects of Nanozyme-Based Glucose Biofuel Cells. Nanomaterials.

[10] Prakobkij A., Saenmuangchin R., Chunta S., Amatatongchai M., Citterio D., Jarujamrus P. (2024). Peroxidase-like Activity of Aptamer-Gold Nanoparticles for Selective and Sensitive Fluorescence Detection of Low-Density Lipoproteins. ACS Appl. Nano Mater..

[11] Singh A.K., Bijalwan K., Kaushal N., Kumari A., Saha A., Indra A. (2023). Oxidase-like Nanozyme Activity of Manganese Metal–Organic Framework Nanosheets for Colorimetric and Fluorescence Sensing of l-Cysteine. ACS Appl. Nano Mater..

[12] Fang G., Kang R., Chong Y., Wang L., Wu C., Ge C. (2023). MOF-based DNA hydrolases optimized by atom engineering for the removal of antibiotic-resistant genes from aquatic environment. Appl. Catal. B Environ..

[13] Lucecki C.A., Durigon D.C., Terenzi H., Bortoluzzi A.J., Neves A., Peralta R.A. (2025). Improving the hydrolase-like activity of a lanthanum (III) complex through second coordination sphere. Inorganica Chim. Acta.

[14] Wang M., Qi W., Wei G., Kumbar S.G. (2020). 16—Assembled peptides for biomimetic catalysis. Artificial Protein and Peptide Nanofibers.

[15] Zhang N., Meng X.-G., Wu Y.-Y., Song H.-J., Huang H., Wang F., Lv J. (2019). Highly Selective Isomerization of Glucose into Fructose Catalyzed by a Mimic Glucose Isomerase. ChemCatChem.

[16] Feng Z., Guo Y., Zhang Y., Zhang A., Jia M., Yin J., Shen G. (2024). Nanozymes: A bibliometrics review. J. Nanobiotechnology.

[17] Ornelas-González A., Rito-Palomares M., González-González M. (2022). TMB vs ABTS: Comparison of multi-enzyme-based approaches for the colorimetric quantification of salivary glucose. J. Chem. Technol. Biotechnol..

[18] Nguyen P.T., Lee J., Cho A., Kim M.S., Choi D., Han J.W., Kim M.I., Lee J. (2022). Rational Development of Co-Doped Mesoporous Ceria with High Peroxidase-Mimicking Activity at Neutral pH for Paper-Based Colorimetric Detection of Multiple Biomarkers. Adv. Funct. Mater..

[19] Nguyen Q.H., Lee D.H., Nguyen P.T., Le P.G., Kim M.I. (2023). Foldable paper microfluidic device based on single iron site-containing hydrogel nanozyme for efficient glucose biosensing. Chem. Eng. J..

[20] Le P.G., Jung S.-C., Han J.-H., Cho S. (2025). Nanozyme-integrated paper chip based on high peroxidase-like activity of Zn-doped reduced graphene oxide for glucose sensing. Microchem. J..

[21] Das B., Lou-Franco J., Gilbride B., Ellis M.G., Stewart L.D., Grant I.R., Balasubramanian P., Cao C. (2022). Peroxidase-Mimicking Activity of Biogenic Gold Nanoparticles Produced from *Prunus nepalensis* Fruit Extract: Characterizations and Application for the Detection of *Mycobacterium bovis*. ACS Appl. Biomater..

[22] Zhang S., Lu Q., Wang F., Xiao Z., He L., He D., Deng L. (2021). Gold–Platinum Nanodots with High-Peroxidase-like Activity and Photothermal Conversion Efficiency for Antibacterial Therapy. ACS Appl. Mater. Interfaces.

[23] Chen L., Yang J., Chen W., Sun S., Tang H., Li Y. (2020). Perovskite mesoporous LaFeO_3_ with peroxidase-like activity for colorimetric detection of gallic acid. Sens. Actuators B Chem..

[24] Dong H., Du W., Dong J., Che R., Kong F., Cheng W., Ma M., Gu N., Zhang Y. (2022). Depletable peroxidase-like activity of Fe_3_O_4_ nanozymes accompanied with separate migration of electrons and iron ions. Nat. Commun..

[25] Wang Y., Feng Q., Liu M., Xue L., Wang G., Zhang S., Hu W. (2023). N, P, S Codoped Carbon Nanozymes with Enhanced Peroxidase-like Activity and Binding Affinity for Total Antioxidant Capacity Assay. ACS Appl. Nano Mater..

[26] Le P.G., Le X.A., Duong H.S., Jung S.H., Kim T., Kim M.I. (2024). Ultrahigh peroxidase-like catalytic performance of Cu–N_4_ and Cu–N_4_S active sites-containing reduced graphene oxide for sensitive electrochemical biosensing. Biosens. Bioelectron..

[27] Yi Y., Zhou X., Liao D., Hou J., Liu H., Zhu G. (2023). High Peroxidase-Mimicking Metal–Organic Frameworks Decorated with Platinum Nanozymes for the Colorimetric Detection of Acetylcholine Chloride and Organophosphorus Pesticides via Enzyme Cascade Reaction. Inorg. Chem..

[28] Yang Q.-Y., Wan C.-Q., Wang Y.-X., Shen X.-F., Pang Y.-H. (2023). Bismuth-based metal-organic framework peroxidase-mimic nanozyme: Preparation and mechanism for colorimetric-converted ultra-trace electrochemical sensing of chromium ion. J. Hazard. Mater..

[29] Chen Y., Rong C., Gao W., Luo S., Guo Y., Gu Y., Yang G., Xu W., Zhu C., Qu L.-L. (2024). Ag-MXene as peroxidase-mimicking nanozyme for enhanced bacteriocide and cholesterol sensing. J. Colloid Interface Sci..

[30] Iravani S., Varma R.S. (2022). MXene-Based Composites as Nanozymes in Biomedicine: A Perspective. Nano-Micro Lett..

[31] Gu Y., Han J., Zhang N., Yan W., Guo Y., Tan H., Yang C., Wang F., Yao H. (2024). Bimetallic Cu@Co-MOFs Mimic Peroxidase for Colorimetric Detection of Glutathione. ACS Appl. Nano Mater..

[32] Sruthi V.P., Senthilkumar S. (2024). Prudently designed Se@fMWCNT as a peroxidase mimicking nanozyme for distinctive electrochemical detection of H_2_O_2_ and glutathione. J. Mater. Chem. C.

[33] Li L., Liu X., Zhu R., Wang B., Yang J., Xu F., Ramaswamy S., Zhang X. (2021). Fe^3+^-Doped Aminated Lignin as Peroxidase-Mimicking Nanozymes for Rapid and Durable Colorimetric Detection of H_2_O_2_. ACS Sustain. Chem. Eng..

[34] Kim M.S., Lee J., Kim H.S., Cho A., Shim K.H., Le T.N., An S.S.A., Han J.W., Kim M.I., Lee J. (2020). Heme Cofactor-Resembling Fe–N Single Site Embedded Graphene as Nanozymes to Selectively Detect H_2_O_2_ with High Sensitivity. Adv. Funct. Mater..

[35] Kim M.S., Cho S., Joo S.H., Lee J., Kwak S.K., Kim M.I., Lee J. (2019). N- and B-Codoped Graphene: A Strong Candidate to Replace Natural Peroxidase in Sensitive and Selective Bioassays. ACS Nano.

[36] Siddiqui A.S., Ahmad M.A., Nawaz M.H., Hayat A., Nasir M. (2019). Nitrogen-doped graphene oxide as a catalyst for the oxidation of Rhodamine B by hydrogen peroxide: Application to a sensitive fluorometric assay for hydrogen peroxide. Microchim. Acta.

[37] Varodi C., Pogăcean F., Coros M., Magerusan L., Stefan-van Staden R.-I., Pruneanu S. (2021). Hydrothermal Synthesis of Nitrogen, Boron Co-Doped Graphene with Enhanced Electro-Catalytic Activity for Cymoxanil Detection. Sensors.

[38] Bhardwaj S.K., Knaus T., Garcia A., Yan N., Mutti F.G. (2022). Bacterial Peroxidase on Electrochemically Reduced Graphene Oxide for Highly Sensitive H_2_O_2_ Detection. ChemBioChem.

[39] Xie J., Cao H., Jiang H., Chen Y., Shi W., Zheng H., Huang Y. (2013). Co_3_O_4_-reduced graphene oxide nanocomposite as an effective peroxidase mimetic and its application in visual biosensing of glucose. Anal. Chim. Acta.

[40] Zhuang J., Midgley A.C., Wei Y., Liu Q., Kong D., Huang X. (2024). Machine-Learning-Assisted Nanozyme Design: Lessons from Materials and Engineered Enzymes. Adv. Mater..

[41] Xuan W., Li X., Gao H., Zhang L., Hu J., Sun L., Kan H. (2025). Artificial intelligence driven platform for rapid catalytic performance assessment of nanozymes. Sci. Rep..

[42] Shen X., Wang Z., Gao X.J., Gao X. (2024). Reaction Mechanisms and Kinetics of Nanozymes: Insights from Theory and Computation. Adv. Mater..

[43] Pan F., Li J., Zhao L., Tuersuntuoheti T., Mehmood A., Zhou N., Hao S., Wang C., Guo Y., Lin W. (2021). A molecular docking and molecular dynamics simulation study on the interaction between cyanidin-3-O-glucoside and major proteins in cow’s milk. J. Food Biochem..

[44] Wang P., Linares-Pastén J.A., Zhang B. (2020). Synthesis, Molecular Docking Simulation, and Enzymatic Degradation of AB-Type Indole-Based Polyesters with Improved Thermal Properties. Biomacromolecules.

[45] Zaaba N.I., Foo K.L., Hashim U., Tan S.J., Liu W.-W., Voon C.H. (2017). Synthesis of Graphene Oxide using Modified Hummers Method: Solvent Influence. Procedia Eng..

[46] Poh H.L., Šaněk F., Ambrosi A., Zhao G., Sofer Z., Pumera M. (2012). Graphenes prepared by Staudenmaier, Hofmann and Hummers methods with consequent thermal exfoliation exhibit very different electrochemical properties. Nanoscale.

[47] Moo J.G.S., Khezri B., Webster R.D., Pumera M. (2014). Graphene Oxides Prepared by Hummers’, Hofmann’s, and Staudenmaier’s Methods: Dramatic Influences on Heavy-Metal-Ion Adsorption. ChemPhysChem.

[48] Anegbe B., Ifijen I.H., Maliki M., Uwidia I.E., Aigbodion A.I. (2024). Graphene oxide synthesis and applications in emerging contaminant removal: A comprehensive review. Environ. Sci. Eur..

[49] Feicht P., Biskupek J., Gorelik T.E., Renner J., Halbig C.E., Maranska M., Puchtler F., Kaiser U., Eigler S. (2019). Brodie’s or Hummers’ Method: Oxidation Conditions Determine the Structure of Graphene Oxide. Chem.—A Eur. J..

[50] Jiříčková A., Jankovský O., Sofer Z., Sedmidubský D. (2022). Synthesis and Applications of Graphene Oxide. Materials.

[51] Qadoos A., Rashid M., Naeem M.N., Jiang Z., Moin M., Babar M. (2025). Bandgap engineering in graphene oxide (GO) via integrating DFT calculations with atmospheric pressure microplasma (AMP) treatment for optoelectronic applications. Hybrid Adv..

[52] De Silva K.K.H., Viswanath P., Rao V.K., Suzuki S., Yoshimura M. (2021). New Insight into the Characterization of Graphene Oxide and Reduced Graphene Oxide Monolayer Flakes on Si-Based Substrates by Optical Microscopy and Raman Spectroscopy. J. Phys. Chem. C.

[53] Lee A.Y., Yang K., Anh N.D., Park C., Lee S.M., Lee T.G., Jeong M.S. (2021). Raman study of D* band in graphene oxide and its correlation with reduction. Appl. Surf. Sci..

[54] Sharma M., Rani S., Pathak D.K., Bhatia R., Kumar R., Sameera I. (2021). Temperature dependent Raman modes of reduced graphene oxide: Effect of anharmonicity, crystallite size and defects. Carbon.

[55] Parpal M., El Sachat A., Sotomayor Torres C.M., Gómez-Romero P., Rueda-García D., Chavez-Angel E. (2024). In situ Raman analysis of reduced-graphene oxide-based electroactive nanofluids. Diam. Relat. Mater..

[56] Wu W., Ranasinghe J.C., Chatterjee A., Huang S. (2024). Recent advances on Raman spectroscopy of graphene: Towards biosensing applications. Mater. Chem. Phys..

[57] Li Z., Deng L., Kinloch I.A., Young R.J. (2023). Raman spectroscopy of carbon materials and their composites: Graphene, nanotubes and fibers. Prog. Mater. Sci..

[58] Wu J., Lin H., Moss D.J., Loh K.P., Jia B. (2023). Graphene oxide for photonics, electronics and optoelectronics. Nat. Rev. Chem..

[59] Ferrari I., Motta A., Zanoni R., Scaramuzzo F.A., Amato F., Dalchiele E.A., Marrani A.G. (2023). Understanding the nature of graphene oxide functional groups by modulation of the electrochemical reduction: A combined experimental and theoretical approach. Carbon.

[60] Khine Y.Y., Wen X., Jin X., Foller T., Joshi R. (2022). Functional groups in graphene oxide. Phys. Chem. Chem. Phys..

[61] Hartmann S.J., Iurchenkova A.A., Kallio T., Fedorovskaya E.O. (2020). Electrochemical Properties of Nitrogen and Oxygen Doped Reduced Graphene Oxide. Energies.

[62] Ruiz-Marizcal J.M., Paez-Ornelas J.I., Fernández-Escamilla H.N., Murillo-Bracamontes E.A., Alonso-Núñez G., Perez-Tijerina E.G., Takeuchi N., Romo-Herrera J.M. (2025). From Graphene Oxide to N-Doped Graphene: Understanding the Doping Process. Adv. Energy Sustain. Res..

[63] Prakash D., Manivannan S. (2021). N, B co-doped and Crumpled Graphene Oxide Pseudocapacitive Electrode for High Energy Supercapacitor. Surf. Interfaces.

[64] Song Y., Qu K., Zhao C., Ren J., Qu X. (2010). Graphene Oxide: Intrinsic Peroxidase Catalytic Activity and Its Application to Glucose Detection. Adv. Mater..

[65] Wang D., Song X., Li P., Gao X.J., Gao X. (2020). Origins of the peroxidase mimicking activities of graphene oxide from first principles. J. Mater. Chem. B.

[66] Yokwana K., Ntsendwana B., Nxumalo E.N., Mhlanga S.D. (2023). Recent advances in nitrogen-doped graphene oxide nanomaterials: Synthesis and applications in energy storage, sensor electrochemical applications and water treatment. J. Mater. Res..

[67] Hu Y., Gao X.J., Zhu Y., Muhammad F., Tan S., Cao W., Lin S., Jin Z., Gao X., Wei H. (2018). Nitrogen-Doped Carbon Nanomaterials as Highly Active and Specific Peroxidase Mimics. Chem. Mater..

[68] Liang D., Yang Y., Li G., Wang Q., Chen H., Deng X. (2021). Endogenous H_2_O_2_-Sensitive and Weak Acidic pH-Triggered Nitrogen-Doped Graphene Nanoparticles (N-GNMs) in the Tumor Microenvironment Serve as Peroxidase-Mimicking Nanozymes for Tumor-Specific Treatment. Materials.

[69] Luo S., Sha M., Tian F., Li X., Fu L., Gu Y., Qu L.-L., Yang G.-H., Zhu C. (2022). Nitrogen and boron co-doped graphene nanoribbons as peroxidase-mimicking nanozymes for enhanced biosensing. Chin. Chem. Lett..

[70] Li D., Fu J., Guo S., Cao J., Liu Z., Zhao R. (2025). Theoretical insights into the peroxidase-like activity of N-doped, B-doped and B/N-Codoped graphene. Chem. Phys. Lett..

[71] Xie X., Zhao Y., Fan Y., Jiang L., Liu W., Yang X. (2024). Multifunctional Fe/Cu Dual-Single Atom Nanozymes with Enhanced Peroxidase Activity for Isoniazid Detection and Levofloxacin Degradation. Langmuir.

[72] Jiao L., Wu J., Zhong H., Zhang Y., Xu W., Wu Y., Chen Y., Yan H., Zhang Q., Gu W. (2020). Densely Isolated FeN_4_ Sites for Peroxidase Mimicking. ACS Catal..

